# Imaging Microanalysis of Materials with a Finely Focused Heavy Ion Probe

**DOI:** 10.6028/jres.093.084

**Published:** 1988-06-01

**Authors:** R. Levi-Setti, J. Chabala, Y. L. Wang

**Affiliations:** The Enrico Fermi Institute and Department of Physics, The University of Chicago, Chicago, IL 60637

## Scanning Analytical Heavy Ion Microscopy at High Lateral Resolution

Liquid metal ion sources (LMIS) in view of the quasi-point-like geometry of their emitting region and confined emission cone possess brightness (~ 10^6^ A cm^−2^ sr^−1^) which is adequate for the realization of high current density (~ 1 A cm^−2^), finely focused (⩾20 nm) probes. A 40 keV scanning ion microprobe, which makes use of a Ga LMIS (UC-HRL SIM) is currently employed in our laboratory to obtain chemical maps of materials in a variety of interdisciplinary applications [1]. The instrument is composed of a two-lens focusing column, a high-transmission secondary ion energy analyzer and transport system, and an RF quadrupole mass filter for secondary ion mass spectrometry (SIMS). In addition, two-channel electron multiplier detectors, overlooking the target region, collect secondary electrons or ions for imaging of the surface topography and material contrast of a sample.

Although a lateral resolution of 20 nm has been attained, sensitivity considerations favor operation at somewhat larger (50–70 nm) probe size. The analytical image resolution is in fact critically dependent on the statistics of the mass analyzed signal [2], which in turn is determined by the rate of material removal by sputtering from the sample surface. Such a rate is proportional to the probe current, which decreases with the square of the probe diameter for chromatic-aberration-limited probes such as those extracted from LMIS. Thus at, e.g., 20 nm probe diameter, only 1–2 pA of probe current are available, the erosion rate is of the order ~ 10^−3^ monolayers/s and probe-size resolution can only be attained for elements of high ionization probability which will provide the highest signal statistics over an acceptable recording time (> 1 count/pixel in a 1024 × 1024 pixel scan for a 512 s acquisition time for the UC-HRL SIM).

In view of the well known range of ionization probabilities (ion fractions) among sputtered atomic species, as well as of sputtering yields, the attainable analytical lateral resolution of finely focused probes becomes target-species dependent due to the above considerations. It also follows that for species difficult to ionize, high resolution SIMS imaging microanalysis is altogether precluded, unless by recourse to postionization techniques [3].

Two examples of applications of the UC-HRL SIM to the study of materials will be illustrated in the present context.

## Imaging Micro-SIMS of Aluminum-Lithium Alloys

SIMS techniques are uniquely suited to the study of Li because of the intense ^7^Li^+^ signal emerging from fast ion bombardment of Li-containing materials. The high resolution imaging capability of the UC-HRL SIM can be fully exploited in this case, as demonstrated in preliminary studies of Al-Li alloys containing up to 12.7 at. *%* Li [4,5], In these important alloys, the ^7^Li^+^ signal is detected with a signal-to-noise ratio ≈ 10^5^, and it is feasible to image and identify grain boundary phases and precipitates in the <100 nm range of dimensions.

Samples of binary Al-Li alloys were solution treated for ~10 mins at 570 °C and water quenched. Some of the samples were then aged to give coarse distributions of either the equilibrium δ (AlLi) phase or a combination of δ and metastable δ′(Al_3_Li). The samples were first mechanically polished and then electropolished to produce a flat surface for SIMS imaging.

**Figure 1:**
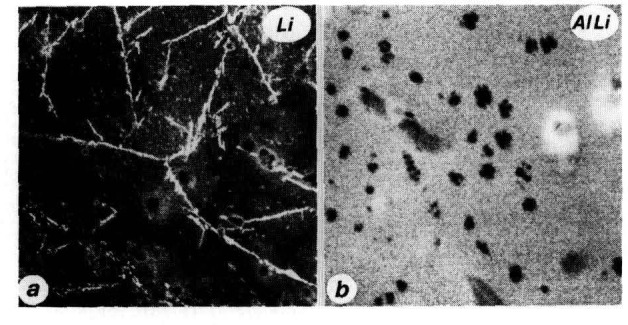
(a) ^7^Li^+^ SIMS image of δ plates in Al-12.7 at. *%* Li alloy. 80 µm full scale, (b) ^34^AlLi^+^ SIMS image of dendritic δ′ particles in Al-10.1 at. % Li. 10 µm full scale.

A number of SIMS artifacts originating in the sample preparation process could be identified and subsequently avoided. These involve Li surface enrichment, surface oxidation and chlorine and carbon contamination. Sputter-cleaning of the samples *in situ* with a 2 kV Ar ion gun was generally necessary to obtain clean and artifact-free surfaces. An example of the detection of δ plates in Al-12.7 at. *%* Li aged 100 hours at 250 °C is shown in the ^7^Li^−^ map of figure 1(a). The plates form in regular crystallographic orientations. A ^34^AlLi^+^ image is shown in figure 1(b) where δ′ particles (dark inclusions) are detected, showing distinctive morphologies that include dendritic growth and nucleation on dislocations. In the latter case, the Al-10.1 at. % Li sample was aged 4 hours at 290 °C.

**Figure 2:**
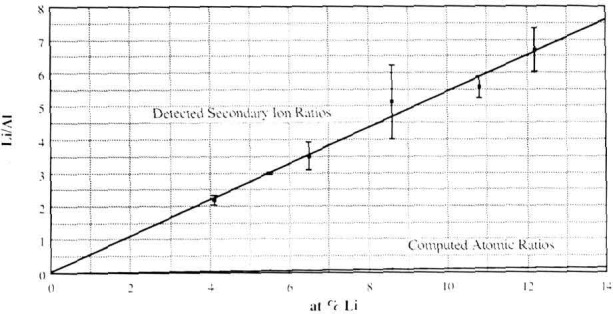
^7^Li^+^/^27^Al^+^ calibration obtained for the UC-HRL SIM from homogeneous as-quenched samples of binary Al-Li alloys.

Toward quantification, a calibration curve has been constructed from ^7^Li^+^/^27^Al^+^ measurements performed on binary Al-Li alloys of known Li concentration. A linear relation between the ^7^Li^+^/^27^A1^+^ ratio and the composition of the standard was observed, as shown in figure 2.

## Imaging Micro-SIMS of Silicon Nitride Ceramics

Sintered silicon nitride is an advanced ceramic often used for high-temperature applications. The sintering process requires the addition of small amounts of oxides in a nitrogen overpressure to prevent dissociation above 1700 °C. Sintering agents such as MgO, Y_2_O_3_ and others, present in 1–10% weight concentrations, react with the silicon nitride phases and the secondary phases, which form along grain boundaries determine the mechanical properties of the ceramic.

Using the UC-HRL SIM, it has been feasible to obtain detailed mapping of the components of the interboundary phases in both yttria- and magnesia-doped silicon nitride [6]. Both fractured and polished surfaces of these ceramics can be readily analyzed in our microprobe, after coating with a thin (~5 nm) Au layer, which is rapidly sputtered away from the field of view under investigation.

**Figure 3:**
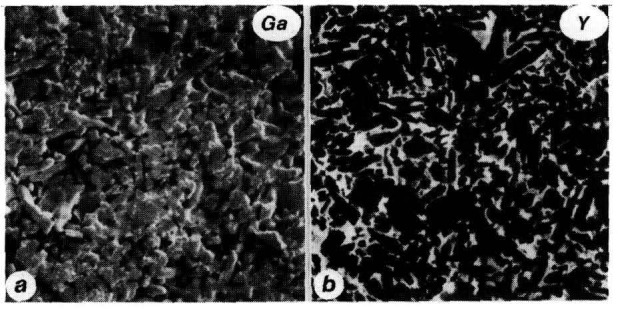
(a) Resputtered ^69^Ga^+^ SIMS image of fracture surface of Y_2_O_3_-sintered silicon nitride ceramic, 20 µm full scale, (b) ^89^Y^+^ image showing distribution of interboundary phase in same area as (a).

In the case of Y_2_O_3_-sintered silicon nitride, the dominant interboundary phase is YSiO_2_N, known to oxidize to Y_2_Si_2_O_7_. SIMS mapping of these phases can be obtained for several break-up fragments such as Y^+^, O^−^, SiO_2_^−^ and SiN^−^. In addition differential resputtering of the implanted Ga^+^ probe ions provides detailed descriptions of the structure of the ceramic comparable to that which can be obtained with backscattered electrons. An example of the level of detail and resolution that can be attained in the imaging microanalysis of this ceramic is shown in figure 3. Figure 3(a) is a ^69^Ga^+^ map, figure 3(b) a ^89^Y^+^ map of the same fracture surface. The silicon nitride crystallites representing the ceramic matrix are clearly outlined in the Ga^+^ map, while the complementary Y^+^ map describes the distribution of the interboundary phase.

Quantification of the composition of the interboundary phase by SIMS is complicated by the presence of matrix effects and local ion yield enhancements in the presence of bound oxygen. Methods are being developed, based on image processing techniques, to perform vector scan microanalysis of either the matrix or the interboundary phases separately. Aided by suitable standards, it is expected that quantification on a microscale may become feasible by this approach.
